# Regulation of SIV Antigen-Specific CD4+ T Cellular Immunity via Autophagosome-Mediated MHC II Molecule-Targeting Antigen Presentation in Mice

**DOI:** 10.1371/journal.pone.0093143

**Published:** 2014-03-26

**Authors:** Yi Jin, Caijun Sun, Liqiang Feng, Pingchao Li, Lijun Xiao, Yizhong Ren, Dimin Wang, Chufang Li, Ling Chen

**Affiliations:** 1 State Key Laboratory of Respiratory Disease, Guangzhou Institutes of Biomedicine and Health (GIBH), Chinese Academy of Sciences, Guangzhou, China; 2 University of Chinese Academy of Sciences, Beijing, China; 3 School of Life Sciences, University of Science and Technology of China (USTC), Hefei, China; 4 School of Life Sciences, Anhui University, Hefei, China; 5 State Key Laboratory of Respiratory Disease, The First Affiliated Hospital of Guangzhou Medical University, Guangzhou, China; Geisel School of Medicine at Dartmouth, United States of America

## Abstract

CD4+ T cell-mediated immunity has increasingly received attention due to its contribution in the control of HIV viral replication; therefore, it is of great significance to improve CD4+ T cell responses to enhance the efficacy of HIV vaccines. Recent studies have suggested that macroautophagy plays a crucial role in modulating adaptive immune responses toward CD4+ T cells or CD8+ T cells. In the present study, a new strategy based on a macroautophagy degradation mechanism is investigated to enhance CD4+ T cell responses against the HIV/SIV gag antigen. Our results showed that when fused to the autophagosome-associated LC3b protein, SIVgag protein can be functionally targeted to autophagosomes, processed by autophagy-mediated degradation in autolysosomes/lysosomes, presented to MHC II compartments and elicit effective potential CD4 T cell responses *in vitro*. Importantly, compared with the SIVgag protein alone, SIVgag-LC3b fusion antigen can induce a stronger antigen-specific CD4+ T cell response in mice, which is characterized by an enhanced magnitude and polyfunctionality. This study provides insight for the immunological modulation between viral and mammalian cells via autophagy, and it also presents an alternative strategy for the design of new antigens in the development of effective HIV vaccines.

## Introduction

Human immunodeficiency virus type-1 (HIV-1) remains a serious challenge, and there are 35.3 million people living with HIV virus and 2.3 million new infections of HIV patients per year worldwide (http://www.unaids.org/en/resources/publications/2013/name,85053,en.asp). However, there is no available HIV vaccine for clinical use, and we do not fully understand the immune correlates of protection against the HIV virus [1]–[3]. HIV-specific cell-mediated immunity, particularly CD8+ T cells, has been recognized in the control of HIV viral replication [4]–[8]. Unfortunately, HIV vaccines based on this rational have failed in recent clinical trials, including STEP [3], [9], [10], Phambili [11] and HVTN505 [3], [12], [13]. Alternatively, antigen-specific CD4+ T cell-mediated immunity has received increased attention due to its contribution in the control of HIV viral replication [14]–[19].

However, immune balance between CD4+ T cells and CD8+T cells might be more important in controlling the HIV virus. One potential reason for the failure of the STEP and HVTN505 trials may be due to the very weak CD4+ T cell response, although a strong CD8+ T cell response was elicited in vaccinees [20], [21]. Recently, data analysis from a phase III trial conducted in Thailand (RV144) showed positive efficacy against HIV acquisition in clinical trials [3], [19], [22], [23], demonstrating that CD4+ T cell-mediated responses, but not CD8+ T cell-mediated responses, were predominantly elicited effectively in vaccinees [24]. Moreover, the CD4+ T cell response was particularly directed to the V2 region of the HIV-1 Env [19]. Interestingly, high levels of V1V2 antibodies were correlated with and contributed to the protection efficacy against HIV-1 infection [24]. Thus, improving the immunogenicity of CD4+ T cell responses for HIV vaccines will be of great significance.

Macroautophagy has been initially studied as a conserved system in the maintenance of cellular homeostasis via the degradation of damaged and long-lived cytoplasmic proteins and organelles via lysosomal machinery in eukaryotic cells [25], [26]. Recently, it has been suggested that macroautophagy plays a crucial role in the regulation of innate and adaptive immune responses [27]–[33]. Furthermore, increased evidence demonstrated that macroautophagy could help to modulate CD4+T cells and/or CD8+ T cells immune responses via the delivery of endogenous peptides, which is usually processed into major histocompatibility complex (MHC) class I, to MHC class II molecules, which usually processes exogenous peptides, a mechanism that is known as “cross-presentation” [34]–[39]. The cytosolic microtubule-associated protein 1 light chain 3 beta (LC3b) is a reliable marker of macroautophagy. LC3b is specifically incorporated into the nascent autophagosome membrane via phosphatidylethanolamine via an ubiquitin-like system, and the autophagosome is then fused with lysosome to become autolysosome. Subsequently, some products of lysosomal degradation are presented on MHC class II molecules and are recognized by CD4+ T cells [33], [40], [41]. Thus, we hypothesized that targeting SIV antigen to autophagosomes might improve CD4+ T cell responses.

In the present study, we investigated this hypothesis using the core structural proteins, group-specific antigen (Gag), of the Simian Immunodeficiency Virus (SIV) as a model antigen. For the first time, our results showed that more robust SIV antigen-specific CD4+ T cellular immune responses, which were characterized by an enhanced magnitude and polyfunctionality, were elicited in mice when SIV antigen was targeted to autophagosomes and MHC class II-containing compartments (MIICs) via fusion to the autophagosome-associated protein LC3b. Taken together, this study provides an alternative strategy in the design of new antigens for effective HIV vaccines.

## Materials and Methods

### Vectors, Antibodies and Peptides

The mouse LC3b gene sequence was obtained from the NCBI data library and chemically synthesized by Invitrogen (USA). The Gag gene sequence was derived from the SIVmac239 virus (SIVgag) and optimized according to the preferred codon usage of mammalian cells as previously described [42]. The N-terminus of LC3b was fused with the C-terminus of SIVgag to obtain the SIVgag-LC3b fusion gene, and SIVgag-LC3b was subsequently cloned into the pVAX1 expression vector (Invitrogen, USA) and adenovirus subtype 5 vectors. Recombinant adenoviruses were generated using homologous recombination methods as previously described [42].

The anti-LC3b antibody was purchased from Sigma. Anti-MHC II (clone M5/114), and anti-LAMP-2 were purchased from Abcam. The anti-SIVgag antibody was kindly provided by the NIH AIDS Research and Reference Reagent Program. Secondary antibodies for immunohistochemistry were Cy3-labeled goat anti-mouse IgG, Alexa Fluor 488-labeled goat anti-mouse lgG, Alexa Fluor 488-labeled goat anti-rabbit IgG or Cy3-labeled goat anti-rat IgG (Beyotime), respectively.

In this study, SIVmac239 Gag peptide pools were obtained from the NIH AIDS Research and Reference Reagent Program. Peptides in this pool were 15 amino acids in length and overlapped by 11 amino acids. All of the peptides were dissolved at 0.4 mg/ml in DMSO.

### Co-culture of DC Cells and CD4+ T cells

Bone marrow-derived dendritic cells (BMDCs) were generated from bone marrow cell cultures as previously described [43]. CD4+ T cells were obtained from C57BL mice, which were immunized with SIVgag antigen, and isolated using anti-CD4 microbeads (Miltenyi). The purified CD4+ T cells were co-cultured with BMDCs. CD4+ T cells were used as effector cells, and BMDCs loaded with different antigens were used as target cells. The ratios were 3∶1 or 9∶1 (E:T). After 48 h, the culture supernatants were measured using an IFN-γ ELISA kit (Dakewei). Mouse IFN-γ at concentrations of 7.8 pg/ml −500 pg/ml was used as a standard. If IFN-γ levels in the supernatants exceeded 500 pg/ml, then the supernatants were diluted and IFN-γ levels were re-measured.

### Animals and Immunization

Six-week-old C57BL/6 female mice were obtained and bred at the Experimental Animals Center of the Guangzhou Institute of Biomedicine and Health (GIBH, Guangzhou, China). The mice were maintained according to the guidelines established by the Association for the Assessment and Accreditation of Laboratory Animal Care. The animal protocol in this study was approved by the Institutional Animal Care and Use Committee of Guangzhou Institute of Biomedicine and Health (permit number 2012039). All procedures were performed by trained personnel and under the supervision of veterinarians. Mice were randomly divided into four groups, and each group consisted of eight mice. Each mouse was intramuscularly injected with 50 μg of the appropriate DNA plasmid in 100 μl PBS at weeks 0 and 2, and then boosted intramuscularly with 1×10^9^ vp (viral particles) of the corresponding adenoviral vector at weeks 4 and 6. At weeks 4 and 8, four mice in each group were sacrificed, and splenocytes were obtained and subjected to subsequent immunological assays.

### Confocal Microscopy

HeLa cells or RAW 264.7 murine macrophage cells were plated and cultured overnight on microscope cover glasses in 12-well plates. Cells were fixed in 4% paraformaldehyde in PBS buffer for 10 min and permeabilized in 0.2% Tween-20 in PBS buffer for an additional 10 min. Next, the cells were blocked for 1 h in 1% bovine serum albumin solution. Corresponding primary and secondary antibodies in 1% bovine serum albumin solution were added and incubated for 60–90 min, followed by three 5-min washes with PBS buffer. Next, the cells were stained with DAPI nucleic acid stain (1 mg/ml, Beyotime) for 3 min. Subsequently, the samples were observed and imaged using an immunofluorescence microscope (LEICA DMI6000B).

### Western Blotting Analyses

To confirm the plasmid expression, HeLa cells were transfected with 1 μg of plasmid using Lipofectamine 2000 (Invitrogen, USA) and lysed with 1% (v/v) Triton X-100 after 48 h. To confirm the adenovirus vector expression, HeLa cells were infected with purified adenovirus virions, collected after 48 h, and then lysed by freeze-thawing three times. Soluble proteins of the cell extracts were subjected to SDS-PAGE and then transferred onto a PVDF membrane (Bio-Rad, Hercules, CA). After blocking for 1 h with 5% skim milk in PBS buffer, the membrane was incubated with anti-LC3b antibody (Sigma) and anti-Gag antibody for 2 h, respectively. The membrane was washed and then incubated with horseradish peroxidase-conjugated goat anti-mouse IgG antibody or anti-rabbit IgG antibody at a 1∶5000 dilution for 1 h. Finally, the specific bands were visualized using enhanced chemiluminescence regents (ECL, GE Amersham).

### IFN-γ ELISPOT Assays

Interferon Gamma (IFN-γ) enzyme-linked immunosorbent spot (ELISPOT) assays were performed as previously described [42], [44]. Briefly, 96-well plates (Millipore, Immobilon-P membrane) were coated with anti-IFN-γ monoclonal antibody (BD Pharmingen) overnight at 4°C and then blocked with 10% fetal bovine serum for 2 h at 37°C. Freshly isolated splenocytes were added at 2×10^5^−8×10^5^ cells/well, and the SIVgag peptides were immediately added at a final concentration of 2 μg/ml. The cells were incubated for 24 h at 37°C, and the expression of IFN-γ was then detected using biotinylated polyclonal anti-mouse IFN-γ (BD Pharmingen) and NBT/BCIP reagent (Pierce). Finally, the number of spots was quantified using an ELISPOT reader (Bioreader4000, BIOSYS, Germany). The data were reported as spot-forming cells (SFCs) per million splenocytes.

### Intracellular Cytokine Staining (ICS)

ICS was processed as previously reported [42], [44]. Briefly, 2×10^6^ freshly isolated mouse splenocytes were stimulated with SIVgag peptides (2 μg/ml per peptide) for 1 h at 37°C. Next, brefeldin A (BD Pharmingen) was added, and the splenocytes were incubated for 16 h at 37°C. The cells were stained with anti-CD3-PerCP, anti-CD4-FITC and anti-CD8-APC monoclonal antibodies (BD Biosciences) for 1 h, permeabilized in FACS Perm buffer for 20 min, and then stained with anti-IFN-γ-PE, anti-TNF-α-PE-cy7, and anti-IL-2-APC-cy7 (BD Pharmingen). Samples were analyzed using the FACSAria instrument with FlowJo software (version 7.6,Tree Star, Inc). The results of the un-stimulated samples were considered to be background and were subtracted from the experimental samples.

### Enzyme-linked Immunosorbent Assay (ELISA)

ELISA assay was performed to detect antibodies against SIVgag (Immune Technology Corp) and LC3b (Abnove) and LC3b proteins. The SIVgag or LC3b proteins were diluted into 1 μg/ml in PBS buffer prior to use. The 96-well plates were coated with 100 μl of protein solution, and incubated overnight at 4°C. The wells were blocked with 200 μl of 5% non-fat powdered milk in PBST for 1 h at 37°C. Diluted serum samples were then added to the plate and incubated for 2 h at 37°C. After washing, goat anti-mouse IgG labeled with horseradish peroxidase (HRP) was added for 1 h. The color reaction was developed by the addition of 100 μl of TMB substrate (Chemicon, Temecula, USA) for 10 min and terminated with 100 μl of 1 M H_2_SO_4._ The absorbance was detected at 450 nm using a Synergy HT Multi-Mode Plate Reader (BioTek Instruments, Inc., Vermont, USA). The binding titer was defined as the reciprocal of the serum dilution at which the absorbance of the test serum was twice that of the negative control serum.

### Data Analysis

Flow cytometric data were analyzed using FlowJo version 7.6 software (Tree Star, Inc., Ashland, OR, USA). Graphical presentations and statistical analyses were performed using the GraphPrism 5.01 software (GraphPad software Inc., La Jolla, CA, USA). A Student’s t-test was performed for comparison of the immune responses between different groups, and a two-tailed p-value of less than 0.05 was considered statistically significant.

## Results

### Generation of Recombinant Constructs Expressing SIVgag-LC3b Fusion Protein

**Figure 1:**
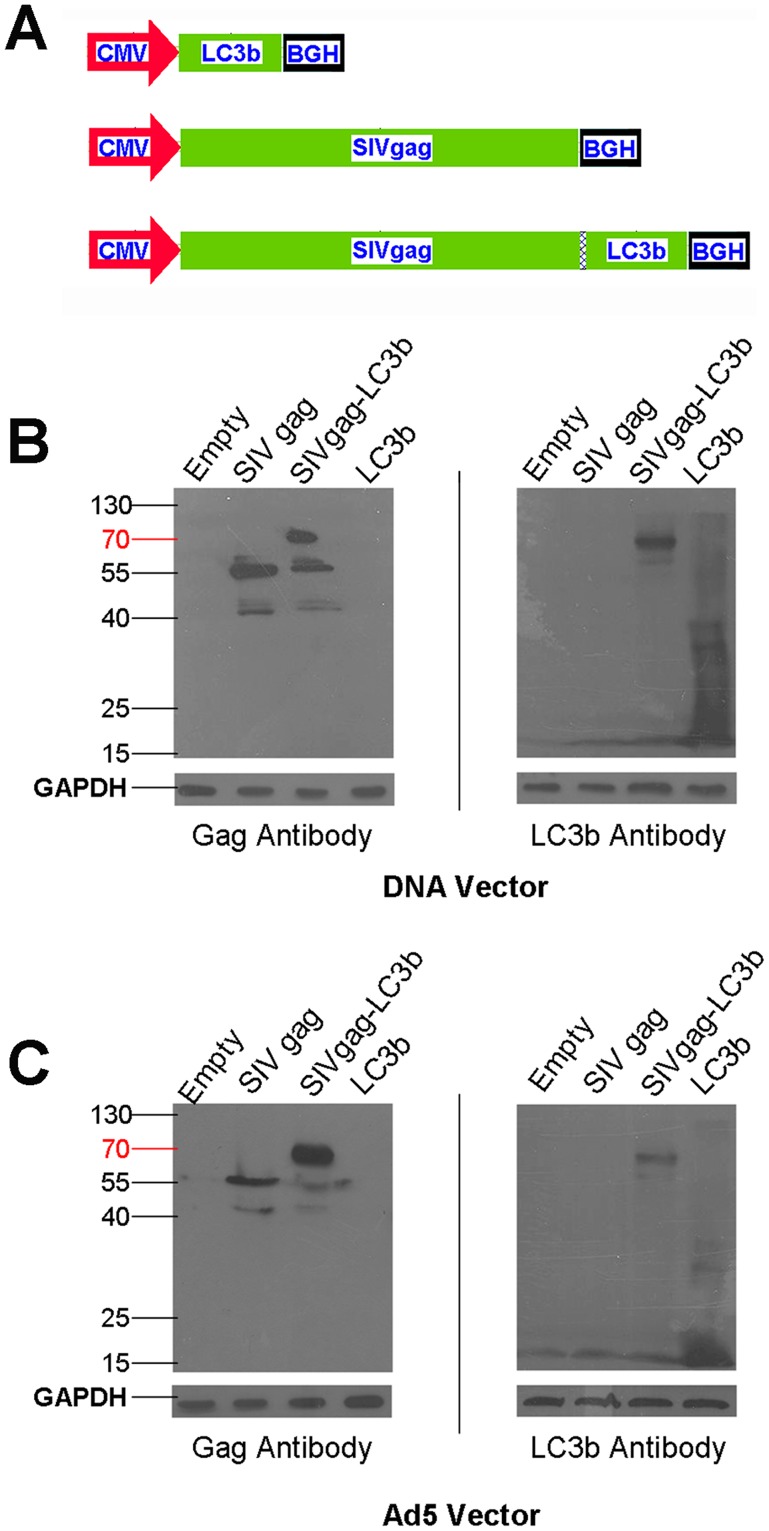
Construction of DNA vectors and Ad5-based vectors carrying the SIVgag-LC3b gene. **A**, Schematic representation of constructs carrying various combinations of mouse LC3b gene or SIVgag under the CMV promoter, denoted as LC3b, SIVgag and SIVgag-LC3b, respectively. HeLa cells were transfected with plasmid-based or Ad5-based constructs, and expression of the protein of interest was detected using western blotting analyses. The GAPDH blot demonstrates equal protein loading. **B**, Expression of the fusion protein using recombinant pVAX plasmid DNA constructs. **C**, Expression of fusion protein using recombinant Ad5-based constructs. The left blots show proteins detected using anti-SIV Gag and the right blots show proteins detected using the anti-LC3b antibody, respectively.

To test our hypothesis, we designed and constructed a series of recombinant DNA vectors and adenoviral vectors carrying various combinations of mouse LC3b gene, SIVgag gene, SIVgag-LC3b fusion gene, respectively (Figure 1A). Expression of the fusion proteins encoded by these recombinant DNA vectors and adenoviral-based vectors were confirmed using western blotting analysis, which revealed that both the DNA constructs (Figure 1B) and adenoviral-based constructs (Figure 1C) expressed the proteins of interest, and exhibited the expected molecular weight (LC3b protein, 16 kD; SIVgag protein, 55 kD; SIVgag-LC3b fusion protein, 71 kD). The SIVgag-LC3b fusion proteins were recognized using either an anti-Gag (Figure 1B and 1C, Left) or anti-LC3b antibody (Figure 1B and 1C, Right).

### Functional Targeting of SIV Gag Protein to Autophagosomes, Lysosomes and MHC II Compartments by Fusing with the LC3b Protein

**Figure 2:**
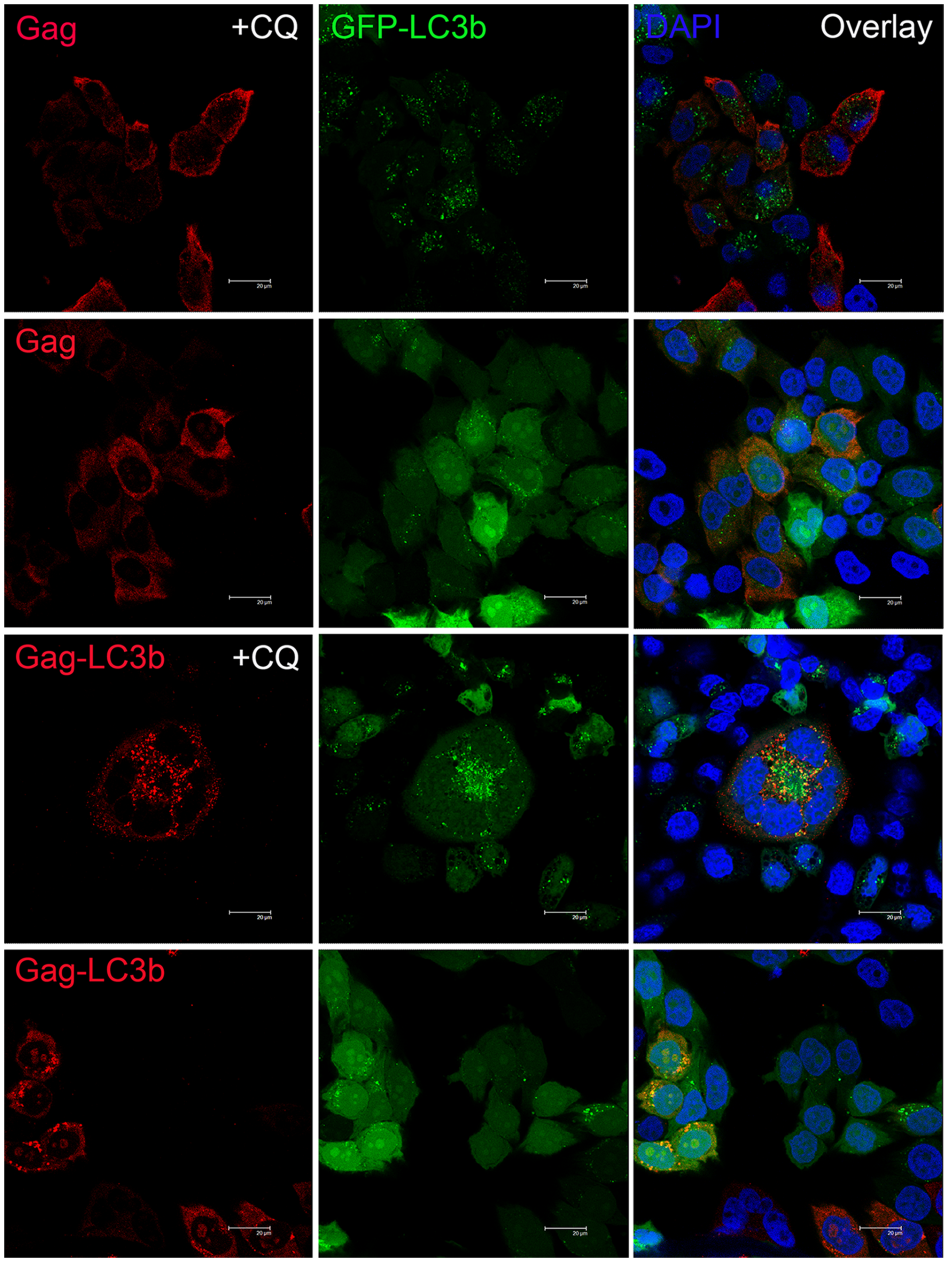
Functionally targeted SIV Gag protein to autophagosomes using confocal microscopy. HeLa-GFP-LC3 cell line stably expressing GFP-LC3 protein (green fluorescence) were transfected with pVAX-SIVgag plasmid (top) or pVAX-SIVgag-LC3 plasmid (bottom) with or without chloroquine (CQ) treatment, and then stained with anti-SIVgag and Cy3-labeled goat anti-mouse IgG as secondary antibodies (red fluorescence); the nuclei were stained with DAPI (blue fluorescence). The scale bar represents 20 μm. Representative cells are shown from one experiment out of three total experiments.

To confirm whether the SIVgag protein could be functionally targeted to autophagosomes, lysosomes and MHC II compartments, we fused the SIVgag protein with LC3b, and observed the subcellular localization of the SIVgag protein in the presence or absence of LC3b protein fusion using confocal microscopy. The formation of autophagosomes is characterized by the redistribution of LC3 protein from a diffuse pattern to a punctate pattern; thus, we generated a HeLa cell line that stably expressed GFP-LC3 protein (HeLa- GFP-LC3 cell line) to visualize autophagosomes. Chloroquine (CQ) treatment can block the fusion of autophagosomes with lysosomes, thereby enabling the visualization of the accumulation of autophagosomes [35]. We found that independent of the presence or absence of CQ, SIVgag protein without LC3b fusion was distributed homogenously throughout the cytoplasm, and the location of the SIVgag protein was different with GFP-LC3 puncta, which are reliable markers of autophagosomes (Figure 2, top). However, when fused with the LC3b protein, the SIVgag-LC3b fusion protein was redistributed in the cytoplasm in a punctate pattern. The amount of both the SIVgag-LC3 puncta and GFP-LC3 puncta were increased with CQ treatment. Moreover, SIVgag-LC3 puncta were co-localized with the GFP-LC3 puncta, which demonstrated that the LC3b protein could target the SIVgag protein to autophagosomes (Figure 2, bottom).

**Figure 3:**
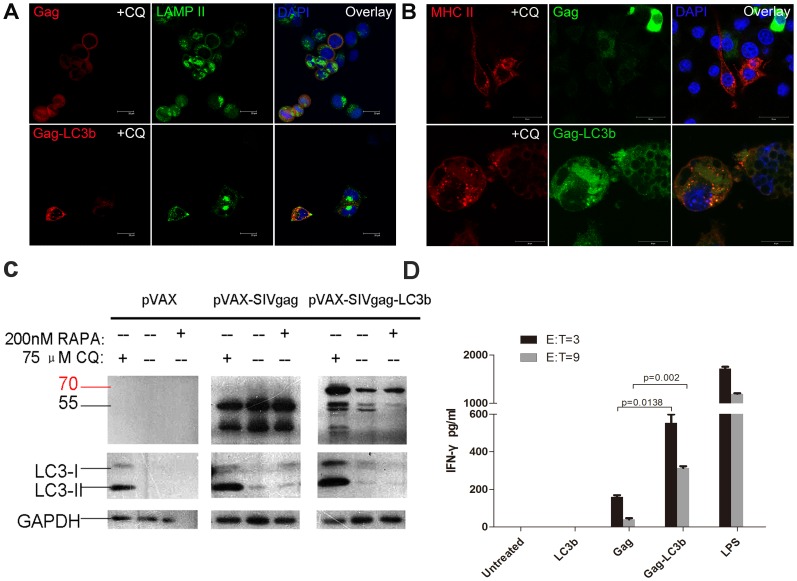
Functionally targeted SIV Gag protein to lysosomes and MHC II compartments using confocal microscopy. **A**, HeLa cells were transfected with pVAX-SIVgag plasmid (top) or pVAX-SIVgag-LC3 plasmid (bottom) with chloroquine (CQ) treatment, and then stained with mouse anti-SIVgag IgG antibody and rabbit anti-LAMP-2 IgG antibody. Subsequently, Cy3-labeled goat anti-mouse IgG (red fluorescence) and Alexa Fluor 488-labeled goat anti-rabbit IgG (green fluorescence) secondary antibodies were used; the nuclei were stained with DAPI (blue fluorescence). The scale bar represents 20 μm. The data represent three independent experiments. **B**, RAW 264.7 cells were stimulated with lipopolysaccharide(LPS) and infected with Ad5-SIVgag (top) or Ad5-SIVgag-LC3 plasmid (bottom) with chloroquine (CQ) treatment, and subsequently stained with mouse anti-SIVgag IgG antibody and rat anti-MHC- II IgG antibody. Next, Cy3-labeled goat anti-rat IgG (red fluorescence) and 488-labeled goat anti-mouse IgG (green fluorescence) secondary antibodies were used; the nuclei were stained with DAPI (blue fluorescence). The scale bar represents 20 μm. The data represent three independent experiments. **C**, HeLa cells were transfected with pVAX, pVAX-SIVgag or pVAX-SIVgag-LC3b for 24 h, and then treated with 75 μM CQ or 200 nM RAPA for 24 h. LC3b-I, LC3b-II and SIVgag were visualized using anti-LC3b and anti-SIVgag immunoblotting, respectively. GAPDH blots demonstrate that CQ or RAPA treatment did not affect the overall protein level. D, SIVgag antigen-specific CD4+ T cells were obtained and isolated using anti-CD4 microbeads, and purified CD4+ T cells (effector cells) were then co-cultured with BMDC cells (target cells). The E:T ratios were 3∶1 or 9∶1. After 48 h, the culture supernatants were measured using the IFN-γ ELISA kit. As a positive control, the BMDC cells stimulated with LPS (1 μg/ml) were used as a positive control, and untreated DMDC cells were used as a negative control. These data were expressed as the mean±SEM from four mice samples.

Subsequently, we confirmed that the SIVgag-LC3b fusion protein was also co-localized to lysosomes using LAMP II, which is a marker of endosomes/lysosomes (Figure 3A). Moreover, the SIVgag-LC3b fusion protein could be co-localized to MHC II compartments in a mouse macrophage cell line, RAW 264.7, when stimulated with lipopolysaccharide (LPS), which up-regulates the expression of MHC class II molecules (Figure 3B). Importantly, CQ treatment did not affect the expression of LAMP II and MHC II molecules in this study (data not shown).

We also observed that the SIVgag-LC3b fusion protein can be processed more effectively via autophagy-mediated degradation compared to SIVgag protein alone. We used rapamycin (RAPA) to induce autophagic flux, and CQ to inhibit the fusion of the autophagosome to the lysosome. Expectedly, we found that when the SIVgag protein was fused with LC3b, its expression level was altered by the induction or inhibition of autophagy. As shown in Figure 3C, SIVgag-LC3b levels were dramatically decreased due to the accelerated degradation via enhanced autophagy with RAPA treatment. Moreover, SIVgag-LC3b was accumulated effectively due to less degradation by the blockade of autophagy with CQ treatment. However, there was no obvious change for the expression level of pVAX-SIVgag when treated with CQ or RAPA.

Taken together, these data demonstrated that the SIVgag-LC3b fusion protein could be functionally targeted to autophagosomes, processed by autophagy-mediated degradation in autolysosomes/lysosomes and presented to MHC II compartments.

### Enhancement of Antigen Presentation to CD4 T cells by Targeting the SIVgag Antigen to Autophagosomes in Dendritic Cells *in vitro*

Given that autophagy in dendritic cells (DCs) has been shown to be important in antigen presentation on MHC-II molecules to CD4 T-cells, we performed a co-culture experiment of DCs and CD4 cells to investigate the efficacy of the SIVgag-LC3b construct to elicit a greater CD4 response. We measured the IFN-γ secretion of antigen-specific CD4+ T cells in response to stimulation with DCs loaded with SIVgag or SIVgag-LC3b protein. Expectedly, compared with DCs loaded with SIVgag protein, DCs loaded with SIVgag-LC3b protein elicited stronger antigen-specific CD4+ T cell responses (Figure 3D). In a lower E:T ratio (3∶1), the SIVgag-LC3b fusion protein elicited approximately 4-fold higher IFN-γ production compared to SIVgag antigen alone (p = 0.0138). In a higher E:T ratio (9∶1), the SIVgag-LC3b fusion protein elicited approximately 10-fold higher IFN-γ production compared to SIVgag antigen alone (p = 0.002). In our study, DCs were stimulated with LPS as a positive control. These data demonstrated that targeting SIVgag antigen to autophagosomes effectively enhanced MHC-II antigen presentation and elicited a greater CD4 T-cell response *in vitro*.

### More Potent Antigen-specific Cellular Immune Responses Elicited by SIVgag-LC3b Fusion Protein Compared to SIVgag Antigen alone *in vivo*

**Figure 4:**
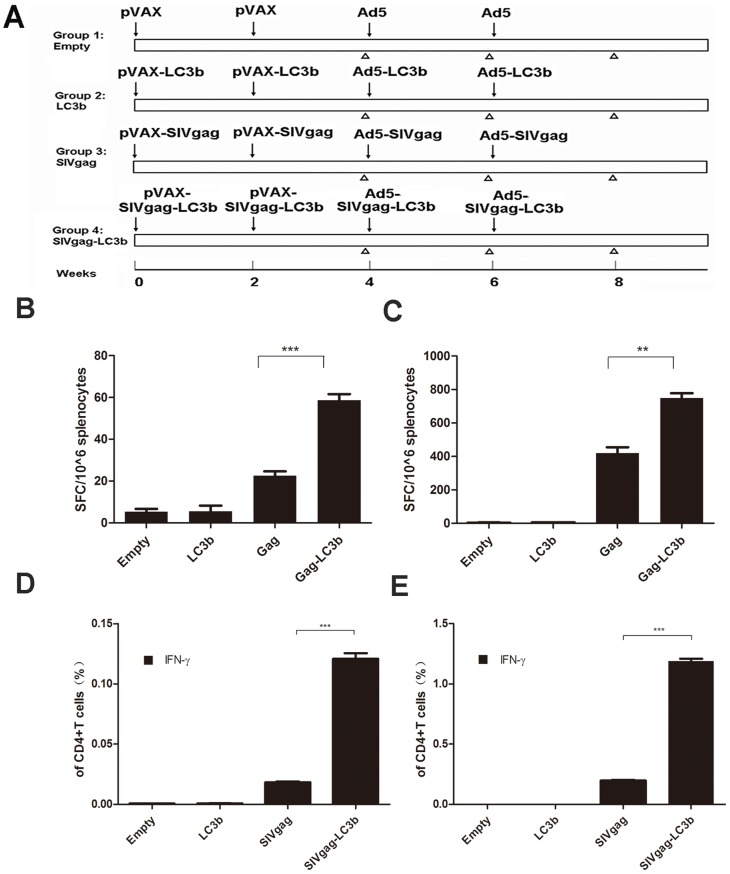
Stronger antigen-specific IFN-γ-secreting CD4 T cell responses elicited by SIVgag-LC3b fusion protein compared to SIVgag antigen alone in mice. **A**, Immunization schedule to evaluate the immunogenicity of the SIVgag-LC3b fusion antigen. C57BL/6 female mice were divided into four groups with 8–10 mice per group. Each mouse was intramuscularly injected 50 μg of the appropriate DNA plasmids at weeks 0 and 2, then boosted intramuscularly with 1×10^9^ vp of corresponding adenoviral vectors at weeks 4 and 6. To assess the immune responses, mice were sacrificed at weeks 4, 6 and 8 after the first immunization to collect splenocytes and serum for analysis of cellular and humoral immune responses. The symbol “↓” represents the time-point of injection; the symbol “**Δ**” represents the time-point of sacrifice and sample collection. The SIVgag-specific cellular immune responses, as assessed using the IFN-γ ELISPOT assay following stimulation with SIVgag peptide, were shown after DNA-based constructs immunization at week 4 (**B**) and after adenoviral-based constructs immunization at week 6 (**C**). The SIVgag-specific cellular immune responses, as assessed using the intracellular IFN-γ cytokine staining assay, were shown after DNA-based constructs immunization at week 4 (**D**) and after adenoviral-based constructs immunization at week 8 (**E**). Data were analyzed using the Student’s t-test, and a two-tailed p-value of less than 0.05 was considered statistically significant. These data were expressed as the mean±SEM from four mice samples (*: p<0.05;**: p<0.01; ***: p<0.001). Two independent experiments for the animal immunization were repeated.

We next aimed to assess whether this fusion protein might induce more potent cellular immunity *in vivo* compared to SIVgag antigen alone. Cohorts of mice were immunized with DNA vectors and Ad5-based vectors expressing the SIVgag protein with or without fusion to the LC3b protein, and the vaccine-elicited, SIVgag-specific cellular immune responses were subsequently monitored using IFN-γ ELISPOT assays. Consistent with our original hypothesis, after plasmid DNA-based prime immunization, the frequency of IFN-γ-secreting cells against SIVgag peptides in the SIVgag-LC3b fusion protein group was significantly higher compared to the group of SIVgag protein alone (Figure 4B, p = 0.0006), and these responses were further enhanced after adenoviral vector-based boost immunization (Figure 4C, p = 0.0019). There was no detectable response against the gag antigen in mock-immunized animals at any time.

Next, we detected the IFN-γ cytokine production by the CD4 T-cells subset. Consistent with the above data, the frequency of SIVgag-specific IFN-γ-secreting CD4 T-cells in the SIVgag-LC3b group was significantly higher compared to the SIVgag group, after plasmid DNA-based prime immunization (Figure 4D, p<0.0001), and these responses were further enhanced after boost immunization (Figure 4E, p<0.0001).

### Amplification of Functional SIVgag-specific CD4+ T and CD8+ T cell Immunity by Fusion with the LC3b Protein

**Figure 5:**
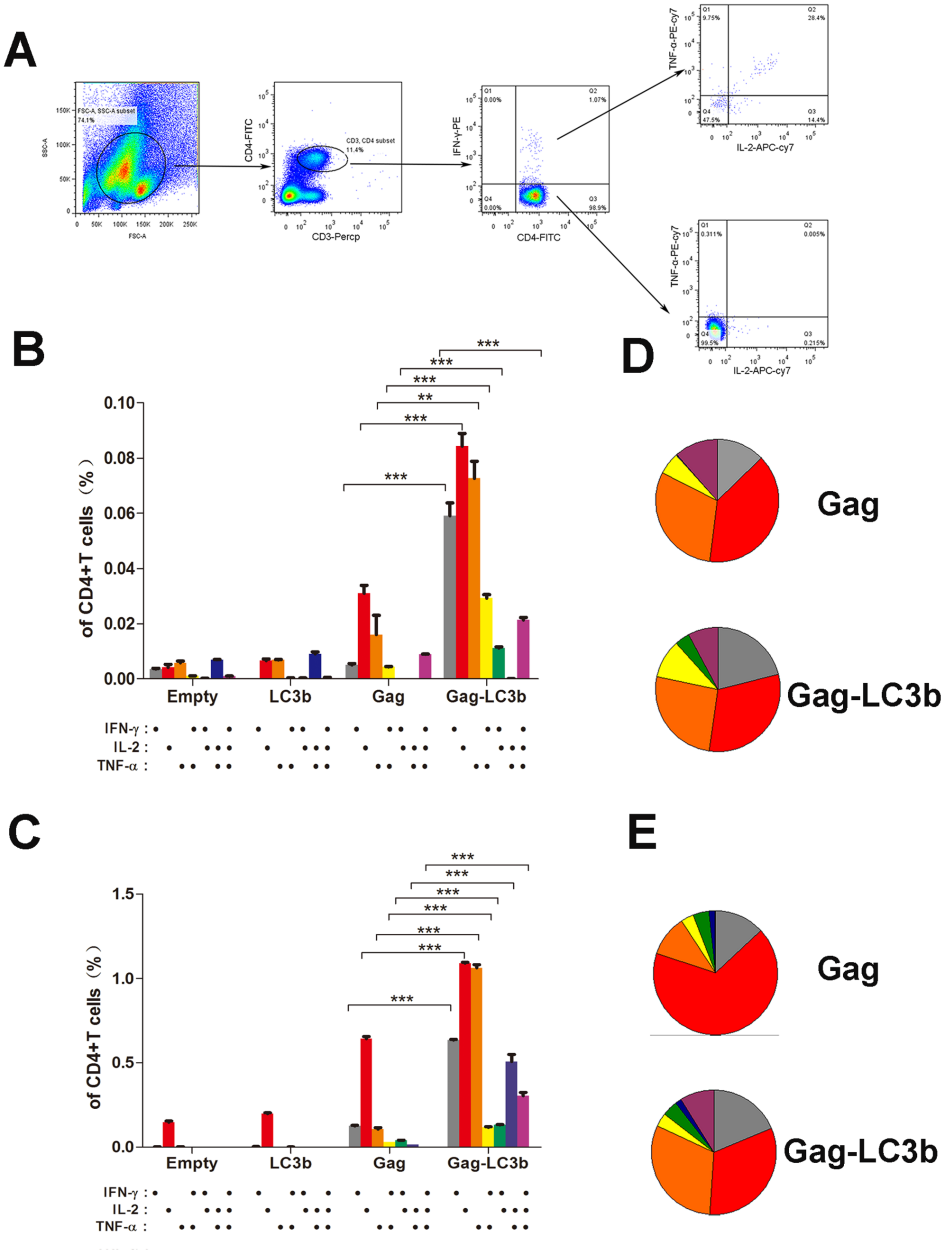
Assessment of polyfunctional SIVgag-specific CD4+ T cellular immunity elicited by the SIVgag-LC3b fusion antigen. Mice were immunized and splenocytes were collected as described in the Materials and Methods and Figure 4A. Splenocytes from four mice in each group were mixed together and 500,000 cells were acquired and analyzed by the FACSAria instrument using the FlowJo software. The ability of functional CD4+ T cell populations from immunized mice to secrete IFN-γ, TNF-α, and IL-2 cytokines in response to SIVgag peptide pool stimulation was assessed. (A) Gating strategy for flow cytometric scatter plots to analyze the frequency of cytokine(s)-positive CD4+ T cells positive in this study. Column graphs depicting subpopulations of single-, double-, or triple-positive CD4+ T cells secreting the cytokines IFN-γ, TNF-α, and IL-2 induced by DNA-based immunization at week 4 (B) or adenoviral-based immunization at week 8 (C). Pie chart analysis was performed to represent subpopulations of cytokine-secreting CD4+ T cells positive for the combination of IFN-γ, TNF-α, and IL-2 induced by DNA-based immunization at week 4 (D) or adenoviral -based immunization at week 8 (E), respectively. The representative data shown here were obtained from two independent experiments from 8–10 mice for each group.

**Figure 6:**
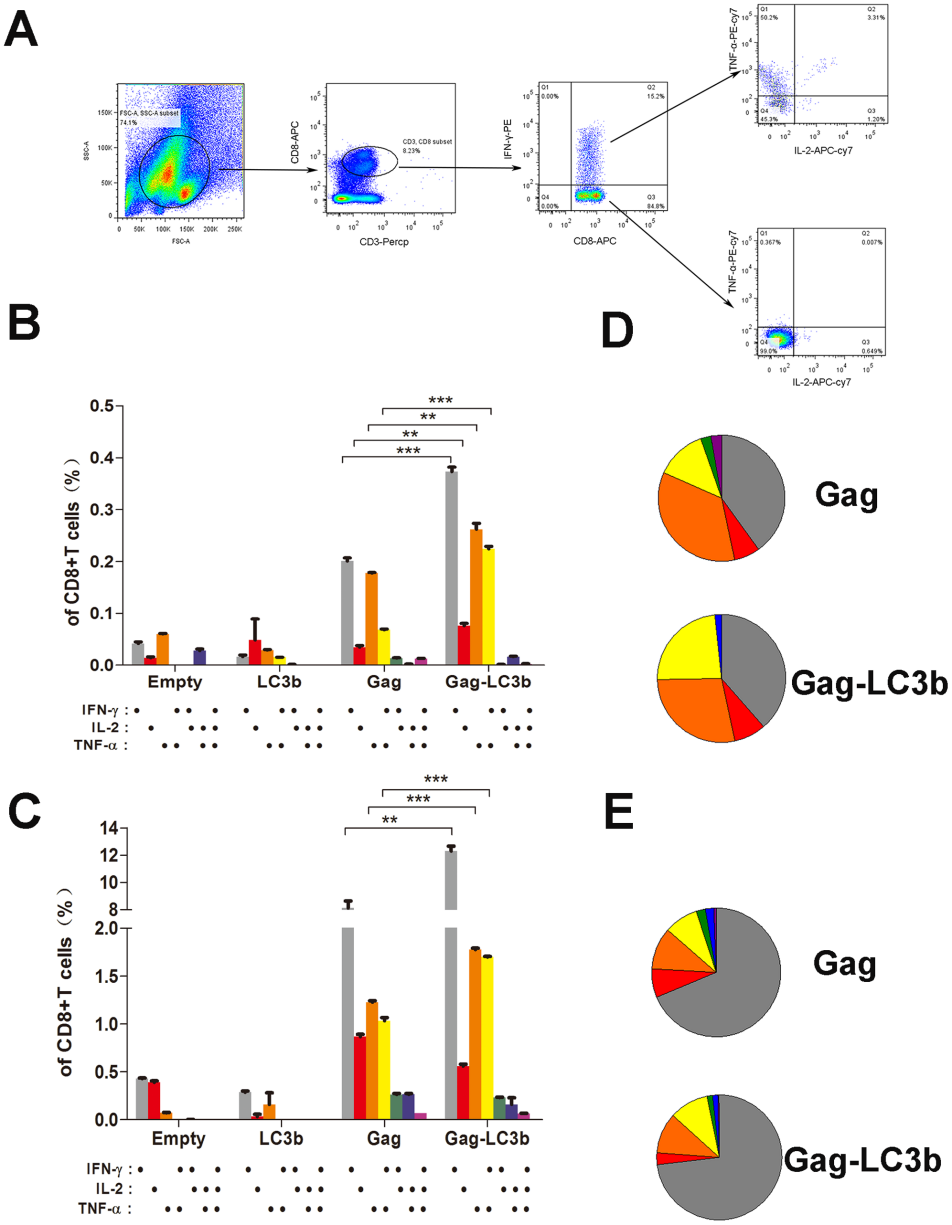
Assessment of polyfunctional SIVgag-specific CD8+ T cellular immunity elicited by the SIVgag-LC3b fusion antigen. Mice were immunized and splenocytes were collected as described in the Materials and Methods and Figure 4A. Splenocytes from four mice in each group were mixed together and 500,000 cells were acquired and analyzed by the FACSAria instrument using the FlowJo software. The ability of functional CD8+ T cell populations from immunized mice to secrete IFN-γ, TNF-α, and IL-2 cytokines in response to the SIVgag peptide pool stimulation was assessed. (A) Gating strategy for flow cytometric scatter plots to analyze the frequency of cytokine(s)-positive CD8+ T cells positive in this study. Column graphs depicting subpopulations of single-, double-, or triple-positive CD8+ T cells secreting the cytokines IFN-γ, TNF-α, and IL-2 induced by DNA-based immunization at week 4 (B) or adenoviral -based immunization at week 8 (C). Pie chart analysis was performed to represent subpopulations of cytokine-secreting CD8+ T cells positive for combinations of IFN-γ, TNF-α, and IL-2 induced by DNA-based immunization at week 4 (D) or adenoviral -based immunization at week 8(E). The representative data shown here were obtained from two independent experiments from 8–10 mice for each group.

Next, we assessed the ability of functional CD4+ T and CD8+ T cell populations from immunized mice to secrete IFN-γ, TNF-α, and IL-2 cytokines in response to SIVgag peptide pool stimulation. CD8+ T or CD4+ T cell subsets production of one or more cytokines (IFN-γ, TNF-α, and IL-2) can be analyzed using the depicted gating strategy (Figure 5A and Figure 6A). After plasmid DNA-based prime immunization, SIVgag-LC3b fusion protein induced a significant higher frequency of SIVgag-specific cytokine(s)-positive both CD4+ T cell subsets (Figure 5B), either IFN-γ alone, TNF-α alone, IL-2 alone, dual IFN-γ/TNF-α, dual FN-γ/IL-2, or triple IFN-γ, TNF-α, and IL-2 compared with SIVgag protein alone. The frequency of SIVgag-specific cytokine(s)-positive CD4+ T cells in SIVgag-LC3b group was 3- to 6-fold more compared to the SIVgag group (Figure 5B). In addition, there was a modestly higher frequency of SIVgag-specific cytokine(s)-positive CD8+ T cell subsets in SIVgag-LC3b group (Figure 6B). The frequency of a single cytokine (IFN-γ alone, TNF-α alone or IL-2 alone) and dual IFN-γ/TNF-α -secreting CD8+ T cells in SIVgag-LC3b group was 2- to 3-fold higher compared to the SIVgag group (Figure 6B).

Interestingly, after boost immunization, the frequency of SIVgag-specific cytokine(s)-positive CD4+ T cells in the SIVgag-LC3b group was persistently elevated with up to a 3- to 10-fold increase compared to the SIVgag group (Figure 5C), either IFN-γ alone, TNF-α alone, IL-2 alone, dual IFN-γ/TNF-α, dual FN-γ/IL-2, dual TNF-α/IL-2, or triple IFN-γ, TNF-α, and IL-2. However, there was merely a 1.5- to 2-fold difference for CD8+ T cells responses between the SIVgag-LC3b group and SIVgag group, which was characterized by CD8+ T cells secreting IFN-γ alone, TNF-α alone and dual IFN-γ/TNF-α (Figure 6C).

In addition, we found that cytokine(s)-positive CD4+ T cells, which were elicited in the SIVgag group, predominantly produced IL-2 and/or TNF-α only, but the CD4 T cells in the SIVgag-LC3b group secreted either IFN-γ alone, TNF-α alone, IL-2 alone, dual IFN-γ/TNF-α, dual FN-γ/IL-2, dual TNF-α/IL-2, or triple IFN-γ, TNF-α, and IL-2 (Figure 5B, 5C, 5D and 5E). Thus, we proposed that more polyfunctional and balanced CD4+ T cell responses were induced against the SIVgag-LC3b fusion protein. However, cytokine(s)-positive CD8+ T cells either in the SIVgag group or SIVgag-LC3b group predominantly produced IFN-γ and/or TNF-α only, and there were no obvious changes for these polyfunctional CD8+ T cell responses (Figure 6B, 6C, 6D and 6E).

### More Robust SIVgag-specific Antibodies Induced by the SIVgag-LC3b Fusion Protein

**Figure 7:**
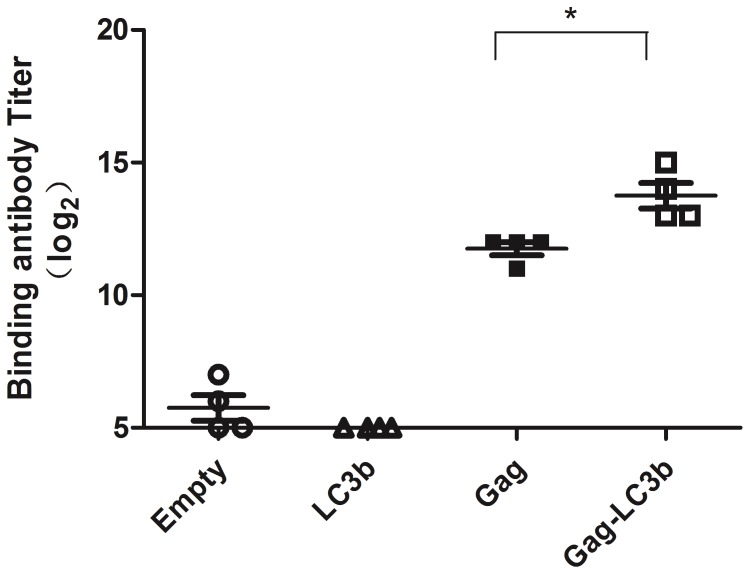
Stronger SIVgag-specific antibodies elicited in mice using the SIVgag-LC3b fusion protein compared to SIVgag antigen alone. Mice were immunized and serums were collected at week 8 as shown in the Materials and Methods. The SIV-binding antibody was assessed using ELISA. Data were analyzed using the Student’s t-test, and a two-tailed p-value of less than 0.05 was considered statistically significant (*: p<0.05). Each data point represents the antibody titer from an individual mouse (n = 4). The representative data were obtained from two independent experiments.

Finally, we detected the humoral responses elicited by the SIVgag-LC3b fusion protein. A significantly higher level of SIVgag-specific antibodies was observed in the group immunized with the SIVgag-LC3b fusion protein compared to the SIVgag protein alone (Figure 7, p = 0.01). Importantly, there was no detectable antibody against the LC3 protein in any animals at any time in this study (data not shown).

## Discussion

Antigen-specific CD4+ T cell-mediated immunity has received increasing attention in the control of HIV viral replication. Herein, we explored a new strategy, based on a degradation mechanism of macroautophagy, to improve CD4+ T cellular immune responses against HIV/SIV antigen. We demonstrated that targeting of the SIV Gag antigen to autophagosomes resulted in an enhanced MHC class II presentation and thus an improved SIVgag-specific CD4+ T cellular immune response.

T lymphocytes of the adaptive immune responses can only recognize the peptide fragment of antigens that are displayed on antigen-presenting cell (APC) surfaces via specialized glycoproteins-the major histocompatibility complex (MHC) molecules. Typically, peptides of endogenous antigens derived from the cytosol and nuclear proteins are bound to MHC class I molecules and are recognized by CD8+ T cells, whereas peptides of exogenous antigens that are generated in lysosomes are bound to MHC class II molecules and recognized by CD4+ T cells. Many antigens derived from viruses, including adenovirus, HIV-1, HCV, among other viruses, could take over the host cell machinery for infection and replication, and are usually processed as endogenous antigens. This can be one reason why dominant CD8+ T cell responses, but weak CD4+ T cell responses, are usually elicited by these viruses and viruses-based vectors. Consequently, it is of great interest to explore a new strategy to regulate immune responses to CD4+ T cells or CD8+ T cells.

Interestingly, recent studies have demonstrated that macroautophagy plays a key role in delivering some endogenous proteins to MHC class II molecules, but not MHC class I molecules, and contribute to CD4+ T cells immune responses [33], [35], [39]. It has been reported that approximately 20%–30% natural MHC class II ligands are derived from endogenous cytosolic proteins [45], [46]. Moreover, it has been reported that the influenza matrix protein 1 fused with autophagosome-associated protein Atg8/LC3B enhanced MHC II presentation to CD4+ T cells [35]. However, there has not yet been a report regarding whether HIV/SIV antigen- specific CD4+ T cellular immunity can be regulated by the autophagosome-mediated MHC II molecules-targeting antigen presentation.

In the present study, we improved the immunogenicity of CD4+ T cell responses for HIV vaccine using a strategy based on macroautophagy degradation. As a proof-of-concept study, we fused the SIVmac239 gag protein to autophagosome-associated protein LC3b (SIVgag-LC3b). Interestingly, we found that this SIVgag-LC3b fusion protein could be functionally targeted to autophagosomes, processed by autophagy-mediated degradation in autolysosomes/lysosomes, presented to MHC II compartments and effectively elicited potential CD4 T cell responses *in vitro*. Importantly, compared with the SIVgag protein alone, the SIVgag-LC3b fusion antigen induced a stronger antigen-specific CD4+ T cell response and humoral immune response *in vivo*. We also observed that the ability of CD4+ T cells to secrete IL-2 cytokine was greatly improved. Because IL-2 is required to stimulate and sustain the proliferation of CD4+ T cells, CD8+ T cells and B cells, and the production of both binding antibodies and neutralizing antibodies is dependent on CD4+ helper T cells, which provide signals for the proliferation and differentiation of B cells [14], [47]–[49]. Thus, it is not surprising that there were also enhanced CD8+ T cell responses and B cell-based antibodies in our study.

Surprisingly, this fusion protein induced potential multifunctional CD4+ T and CD8+ T cell populations secreting IFN-γ, TNF-α, and IL-2 cytokines in response to SIVgag peptide pool stimulation, particularly for CD4+ T cell subsets to produce dual or triple types of IFN-γ, TNF-α, and IL-2 cytokines. The conflicting roles of CD4+ T cells as both immune effectors and targets for the HIV virus may be one concern for our study. It has been proposed that CD4+ T cells are a double-edged sword in HIV infection and protection. From one aspect, it is well known that CD4+ T cells, including memory CD4+ T cells in the intestinal mucosa, are the main target of HIV-1. During acute HIV-1 infection, a massive loss of memory CD4+ T cells occurs throughout the body [50], [51]. However, from another aspect, increasing data have shown that the ability to induce functional antigen-specific CD4+ T subsets is extensively correlated with immune protection against HIV infection [14], [52], [53]. In particular, the RV144 trial demonstrated that CD4+ T cell-mediated responses, but not CD8+ T cell-mediated responses, were predominantly effectively elicited in vaccinees, and the CD4+ T cell response correlates and contributes to protection efficacy against HIV-1 infection. On the basis of this recent evidence, a potentially effective HIV vaccine should positively strengthen the ability of CD4 T cells to kill the HIV virus. In the present study, we induced potent multifunctional CD4+ T responses. Thus, it is reasonable to expect that an HIV/SIV vaccine based on this novel strategy would be promising, although further work is required to confirm whether this fusion protein could afford effective immune control against pathogenic HIV/SIV infection and replication in the macaque model and clinical trials.

In the present study, plasmid DNA vector is preferred as a model vector to investigate our hypothesis, and immune responses induced by a DNA vector carrying the SIVgag-LC3b fusion gene is promising (Figure 4B, 5B, 6B). However, one concern for this study might be that the adenoviral-based vector is used as another model vector because the choice of the adenoviral vector for HIV vaccine has been extensively debated in view of the unimpressive results obtained with STEP [3], [9], [10] and subsequently with HVTN505 [3], [12], [13], an HIV vaccine clinical trial. However, adenoviral-based vectors usually induce a very strong CD8+ T cell response, but a very weak CD4+ T cell response, which might underlie these failed clinical trials [20], [21]. However, the adenoviral vector expressing the SIVgag-LC3b fusion protein can elicit potential multifunctional CD4+ T cell responses in our study (Figure 5). Thus, we believe that our strategy of antigen modification is effective in regulating immune responses toward CD4+ T cells, although this fusion antigen was expressed by CD8+ T-biased adenoviral vector. Hopefully, it will be better to confirm the immunogenicity of this fusion antigen expressed using others vectors in the future.

Both safety and efficacy are critical concerns when assessing any vaccination or gene therapy, particularly in clinical applications. LC3 protein is extensively expressed in mammalian cells and plays a vital physiological function. For example, LC3 protein is C-terminally conjugated with the autophagosomal double-membrane and comprises the autophagosomes, in which macroautophagy helps the cells to respond to a wide range of extracellular and intracellular stresses, including nutrient starvation, the presence/absence of insulin and other growth factors, among others [25], [26]. As a result, one concern for this immunization strategy might be whether a response against LC3 protein could be unexpectedly induced, while this fusion protein could induce robust immune responses against SIVgag protein. These were some considerations in this study. Intriguingly, there was no detectable cellular response or antibody against LC3 protein in any animal at any time.

Taken together, to the best of our knowledge, this was the first study to report when SIV antigen was fused to autophagosome-associated protein LC3b, this fusion protein could be targeted to autophagosomes and induces potent SIV antigen-specific CD4+ T cellular and humoral immune responses with enhanced magnitude and polyfunctionality in mice. This study will help to provide insight for the understanding of the immunological modulation between viruses and mammalian cells via autophagy. This novel strategy should be further developed as an alternative strategy to design new antigens for an effective vaccine against HIV, other infectious diseases and cancer.
